# Abnormal inter-ventricular diastolic mechanical delay in patients with ST-segment elevation myocardial infarction

**DOI:** 10.1186/s12872-023-03531-1

**Published:** 2023-10-06

**Authors:** Wenying Jin, Chao Yu, Lan Wang, Yuliang Ma, Dan He, Tiangang Zhu

**Affiliations:** Department of Cardiology, Beijing Key Laboratory of Early Prediction and Intervention of Acute Myocardial Infarction, Center for Cardiovascular Translational Research, Peking University People’s Hospital, Beijing, China

**Keywords:** Myocardial infarction, Left ventricle, Mechanical synchrony, Diastolic ventricular mechanical delay, Global longitudinal strain, Left ventricular myocardial work, Pulsed-wave Doppler echocardiography

## Abstract

**Background:**

This study aimed to investigate the ventricular mechanical relaxation pattern and its clinical influence in patients with ST-segment elevation myocardial infarction (STEMI).

**Methods:**

Echocardiography was performed to measure mitral and tricuspid diastolic opening times. Left ventricular diastolic mechanical delay (LVMDd) was defined as diastolic filling of the right ventricle earlier than that of the left ventricle, and right ventricular diastolic mechanical delay (RVMDd) was defined as the right ventricular diastolic filling later than left ventricular filling.

**Results:**

Among 152 patients with STEMI, 100 (65.8%) had LVMDd, and 47 (30.9%) had RVMDd. In-hospital complications were significantly increased in patients with RVMDd (61.6% vs. 41.0%, *P* = 0.017). Those with RVMDd exhibited significantly lower left ventricular global longitudinal strain (11.7 ± 4.1% vs. 13.2 ± 4.0%, *P* = 0.035), global work index (913.8 ± 365.9 vs. 1098.9 ± 358.8 mmHg%, *P* = 0.005) and global constructive work (1218.6 ± 392.8 vs. 1393.7 ± 432.7 mmHg%, *P* = 0.021). Mitral deceleration time significantly decreased (127.4 ± 33.5 vs. 145.6 ± 41.7 ms, *P* = 0.012), and the ratio of early mitral inflow to early mitral annular velocity (E/E’) significantly increased [13.0(11.0–20.0) vs. 11.9(9.3–14.3), *P* = 0.006] in the RVMDd group. Logistic regression analysis showed that age (odds ratio [OR]:0.920; *P* = 0.001), brain natriuretic peptide level (OR: 1.1002; *P* = 0.036) and mitral E/E’ (OR: 1.187; *P* = 0.003) were independently associated with RVMDd.

**Conclusions:**

Delayed right ventricular filling is related to more severe left ventricular systolic and diastolic dysfunction in STEMI patients. More attention should be paid to patients with RVMDd to prevent adverse events during hospitalization.

## Introduction

Ventricular mechanical dyssynchrony is a major contributor to the deterioration of heart failure (HF) [1]. Cardiac resynchronization therapy (CRT) has proven to be a successful way to improve hemodynamics, symptoms, and prognosis in patients with HF [2, 3]. Previous studies had mainly focused on the ventricular contraction pattern, proving that ventricular contractile dyssynchrony is associated with adverse cardiovascular outcomes such as HF in patients after myocardial infarction [4–6]. However, few studies had explored diastolic ventricular synchrony.

Previous research has shown that Doppler echocardiography-measured inter-ventricular mechanical dyssynchrony can help predict prognosis in ischemic and non-ischemic dilated cardiomyopathies [7, 8], acute myocardial infarction [4], and coronary artery disease [9]. Our previous study [10] had proved that diastolic ventricular relaxation sequence in normal hearts was highly consistent, with the right ventricle filling before the left ventricle. This indicated that diastolic opening time of the tricuspid value (Q-TV_E_) was less than that of the mitral valve (Q-MV_E_), and we defined this differences as left ventricular diastolic mechanical delay (LVMDd) [10]. However, in patients with HF, the percentage of right ventricular diastolic mechanical delay (RVMDd) increased significantly [10]. This study aimed to explore further the ventricular mechanical relaxation pattern and its influences on patients with ST-segment elevation myocardial infarction (STEMI). Myocardial contrast echocardiography (MCE) and two-dimensional speckle tracking echocardiography (STE) were performed to evaluate left ventricular function accurately.

## Materials and methods

### Study population

We consecutively enrolled patients with STEMI who had completed the MCE examination within 48 h after percutaneous coronary intervention (PCI) between June 2016 and July 2022 in our hospital. Demographic information, medical history, and clinical data were collected and retrospectively analyzed. Patients younger than 18 years or those with known congenital heart disease, significant valvular heart disease, paced rhythm, significant variation in R-R intervals, poor image quality, or missing important echocardiographic data were excluded from the study. The local ethics committee approved this study, and informed consent was obtained from all participants.

### Echocardiographic examination

Pulsed-wave Doppler echocardiography was performed in all participants using an M5S 3.5-MHz transducer (GE Vivid E9, GE Vingmed, Horten, Norway) according to the guidelines of the American Society of Echocardiography [11]. The participants were placed in a left supine position, breathing quietly, and connected to a synchronous electrocardiogram monitor. The readers were blinded to the clinical information. All images were saved in a digital format for subsequent offline analysis using EchoPAC version 203 software (GE Vingmed Ultrasound).

**Fig. 1:**
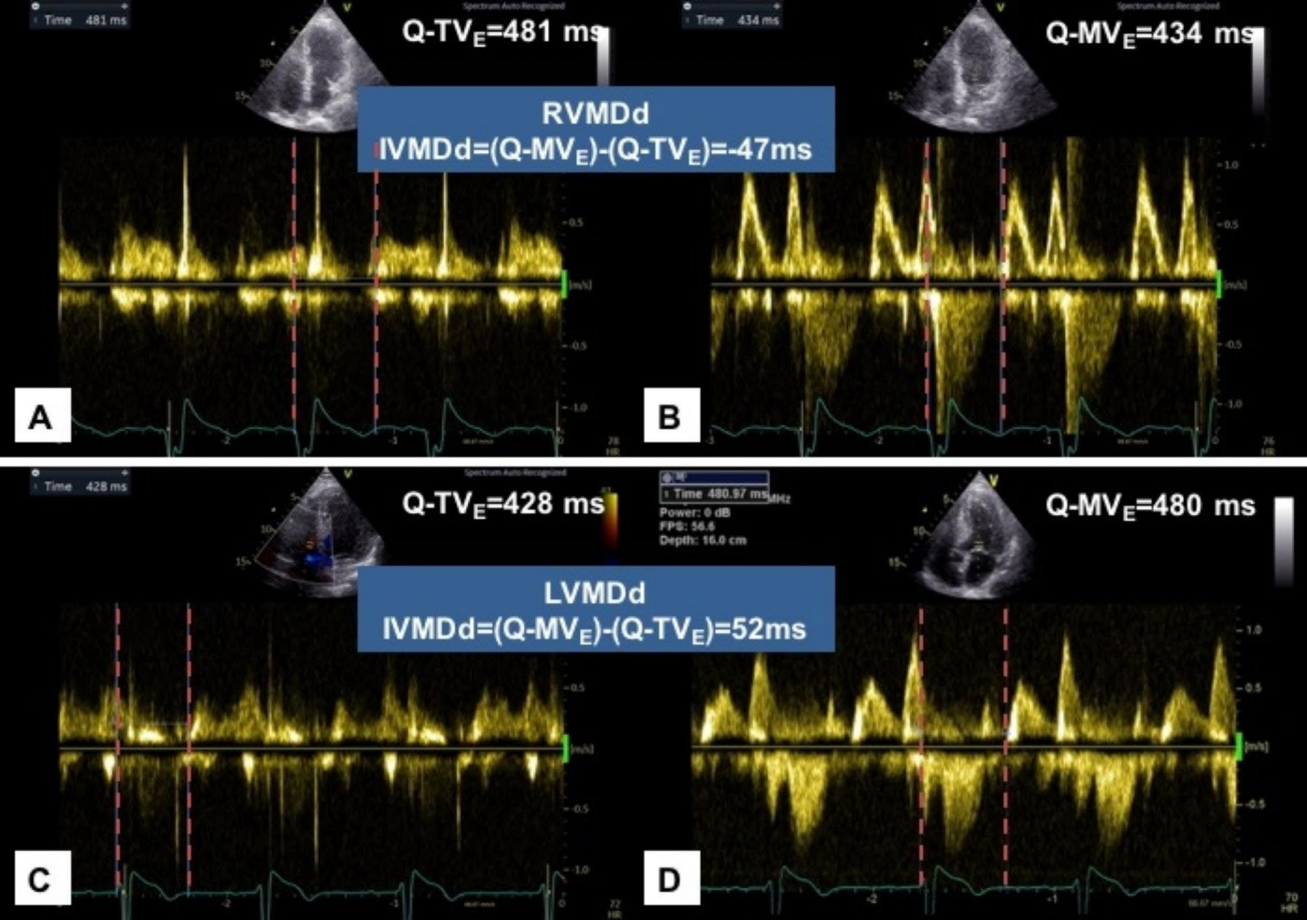
Measurements of IVMDd in STEMI patients. Mechanical sequences between left ventricle and right ventricle in diastole in two patients with STEMI. In the patient with RVMDd (**A,B**), LV filling occurred 47 ms prior to RV filling. In the patient with LVMDd (**C,D**), RV filling occurred 52 ms prior to LV filling. IVMDd, inter-ventricular mechanical delay in diastole; LVMDd, left ventricular diastolic mechanical delay; RVMDd, right ventricular diastolic mechanical delay; STEMI, ST-segment elevation myocardial infarction

As mentioned in a previous study [10], the inflow spectrums of the pulsed-wave Doppler across the mitral and tricuspid valve were recorded in apical four-chamber views. The diastolic opening times of the left and right ventricle were measured as the interval between the onset of the QRS complex and the beginning of the E wave for the mitral valve (Q-MV_E_) and tricuspid valve (Q-TV_E_), respectively (Fig. 1). LVMDd was defined as Q-MV_E_ > Q-TV_E,_ and RVMDd was defined as Q-MV_E_ < Q-TV_E_. Pulsed-wave Doppler across the aortic and pulmonary valves was also recorded to obtain the left ventricular pre-ejection period (LV_PEP_) and right ventricular pre-ejection period (RV_PEP_). Inter-ventricular mechanical delay (IVMD) was defined as the time difference between left ventricular (LV) and right ventricular (RV) mechanical delays. IVMD was positive if RV activation preceded LV activation and negative if LV activation preceded RV activation.

STE was performed to analyze LV global longitudinal strain (GLS) based on three standard apical views (apical long axis, four-chamber, and two-chamber). Left ventricular myocardial work (LVMW), including four indices: global work index (GWI), global constructive work (GCW), global wasted work (GWW), and global work efficiency (GWE), was calculated from LV pressure-strain analysis using EchoPAC version 203 software (GE Vingmed Ultrasound), as previously described [12, 13]. The left ventricular ejection fraction (LVEF) and left ventricular end-diastolic volume index (LVEDVi) using the modified Simpson biplane method were measured by real-time MCE using the enhanced contrast agent sulfur hexafluoride (SonoVue) (Bracco, Italy). Right ventricular functional assessments including tricuspid annular plane systolic excursion (TAPSE), peak systolic myocardial velocity (RVS’) and RV myocardial performance index (RVMPI) were taken as well. Diastolic function variables including mitral deceleration time (EDT), the peak mitral annulus velocity during early diastole (averaged septal and lateral E’) and E/E’ ratio were also collected.

### Statistical analysis

All statistical analyses were performed using SPSS software (Version 26.0, Chicago, IL, USA). Continuous variables were described as means ± standard deviations (SD) for normally distributed variables and as medians (interquartile ranges) for non-normally distributed variables. Categorical variables were described as numbers (percentages). Inter-group comparison was analyzed by Pearson chi square test, independent t-test and Mann Whitney U test. Logistic regression analysis was performed to test variables associated with RVMDd. All *P*-values were two-tailed and a significance level of < 0.05 was used.

## Results

### Distribution patterns of ventricular mechanical delays

A total of 191 patients with STEMI were enrolled consecutively in this study, and data on Q-TV_E_ and Q-MV_E_ were available for 152 patients (range: 23–89 years; mean age:59 ± 13 years, 124 males, 28 females). Mechanical delays of left and right ventricular contraction and relaxation were measured using Doppler echocardiography. LVMDd was found in 100 (65.8%) patients, RVMDd was found in 47 (30.9%) patients, and five patients showed synchronous left and right ventricular relaxation.

Table 1 presented the echocardiographic measurements of the LV and RV mechanical delays in systole and diastole. The systolic pre-ejection period (LV_PEP_, RV_PEP_) showed no difference between RVMDd and LVMDd patients. But the diastolic opening time of mitral valve (Q-MV_E_) in RVMDd group was much shorter than that in LVMDd group (412.0 ± 40.8 ms vs. 444.2 ± 35.8 ms, *P* < 0.001). The interval time of Q-TV_E_ in RVMDd group was much longer than that in LVMDd group (442.3 ± 52.2 ms vs. 404.0 ± 47.0 ms, *P* < 0.001).

**Table 1:** Ventricular mechanical delays

	RVMDd (n = 47)	LVMDd (n = 100)	*P*
Systole (ms)			
LV_PEP_	86.8 ± 16.4	89.2 ± 17.8	0.435
RV_PEP_	92.9 ± 25.1	94.2 ± 33.1	0.812
IVMDs	-6.1 ± 3.2	-4.4 ± 3.2	0.749
Diastole (ms)			
Q-MV_E_	412.0 ± 40.8	444.2 ± 35.8	0.000
Q-TV_E_	442.3 ± 52.2	404.0 ± 47.0	0.000
IVMDd	-30.4 ± 6.3	40.2 ± 4.4	0.000

IVMDd, diastolic inter-ventricular mechanical delay; IVMDs, systolic inter-ventricular mechanical delay; LV_PEP_, left ventricular pre-ejection period; Q-MV_E_, time interval from the onset of QRS complex to the onset of early diastolic E wave of mitral valve; Q-TV_E_, time interval from the onset of QRS complex to the onset of early diastolic E wave of tricuspid valve; RV_PEP_, right ventricular pre-ejection period

### Patient clinical characteristics

Table 2 summarized the basic clinical characteristics of the LVMDd and RVMDd groups. There were no differences in gender, age, or previous medical history between the two groups, except that the prevalence of hypertension was lower in the RVMDd group (53.2% vs. 70.0%, *P* = 0.047). Body mass index (BMI) was significantly increased in the RVMDd group (26.9 ± 3.7 vs. 25.4 ± 3.4, *P* = 0.015). The patients in the RVMDd group had significantly lower systolic blood pressure (SBP, 113 ± 21 mmHg vs. 121 ± 20 mmHg; *P* = 0.037), lower incidence of Killip classification I (78.7% vs. 91.0%; *P* = 0.039), higher values of troponin I (TnI) peak [80.5 (32.9–134.4) ng/mL vs. 51.4 (16.2–107.2) ng/mL; *P* = 0.032] and higher levels of brain natriuretic peptide (BNP) [363.0 (135.5–619.5) pg/mL vs. 190.0 (85.3–437.8) pg/mL; *P* = 0.017] at admission when compared with those in the LVMDd group. There was no difference in the pre and post-PCI data, culprit vessels, or thrombolysis in myocardial infarction blood flow. However, the incidence of total complications in the hospital, including acute HF, arrhythmia, stroke, mechanical complications, use of circulatory support devices or ventilators, re-infarction, and death, significantly increased in the RVMDd group (61.6% vs. 41.0%, *P* = 0.017).

**Table 2:** Clinical characteristics at baseline

	RVMDd (n = 47)	LVMDd (n = 100)	*P*
Age (years)	56 ± 13	61 ± 13	0.053
Male, n(%)	41(87.2)	79(79.0)	0.484
BMI (kg/m^2^)	26.9 ± 3.7	25.4 ± 3.4	0.015
Smoker, n(%)	34(72.3)	63(63.0)	0.265
Hypertension, n(%)	25(53.2)	70(70.0)	0.047
Diabetes, n(%)	16(34.0)	38(38.0)	0.643
Chronic kidney disease, n(%)	4(8.5)	13(13.0)	0.427
Previous myocardial infarction, n(%)	3(6.4)	2(2.0)	0.172
SBP at admission (mmHg)	113 ± 21	121 ± 20	0.037
DBP at admission (mmHg)	75 ± 15	76 ± 15	0.556
Heart rate at admission (bpm)	79 ± 21	78 ± 16	0.841
Killip classification I, n(%)	37(78.7)	91(91.0)	0.039
Symptom onset to balloon time (h)	10.8(6.1–27.7)	13.0(5.5–59.1)	0.660
Maximum troponin I (ng/ml)	80.5(32.9-134.4)	51.4(16.2-107.2)	0.032
CRP (mg/L)	3.1(0.5–28.6)	2.6(0.5–17.5)	0.492
BNP (pg/ml)	363.0(135.5-619.5)	190.0(85.3-437.8)	0.017
Serum creatinine (µmol/L)	77.0(64.0–93.0)	83.5(67.3-104.3)	0.129
Angiographic data			
Three-vessel disease, n(%)	17(36.2)	50(41.7)	0.116
Final TIMI flow ≤ 2, n(%)	8(17.0)	16(16.7)	0.957
Time to PCI (h)	10.8(3.0-287)	13.0(2.0-280)	0.660
LAD STEMI, n(%)	28(59.6)	45(45.0)	0.099
RCA STEMI, n(%)	13(27.7)	42(42.0)	0.094
LCx STEMI, n(%)	6(12.8)	13(13.0)	0.969
Total complications, n(%)	29(61.7)	41(41.0)	0.017
Acute heart failure	7(14.9)	7(7.0)	0.128
Arrhythmia	16(34.0)	24(24.0)	0.170
Mechanical complication	0(0.0)	1(1.0)	0.492
Re-infarction	0(0.0)	3(3.0)	0.230
IABP/ECMO	2(4.3)	1(1.0)	0.193
Ventilators	4(8.5)	3(3.0)	0.143
Stroke	0(0.0)	1(1.0)	0.492
Death	0(0.0)	1(1.0)	0.492

BMI, body mass index; BNP, brain natriuretic peptide; CRP, C-reactive protein; DBP, diastolic blood pressure; LAD, left anterior descending; LCx, left circumflex artery; LVMDd, left ventricular diastolic mechanical delay; PCI, percutaneous coronary intervention; RCA, right coronary artery; RVMDd, right ventricular diastolic mechanical delay; SBP, systolic blood pressure; STEMI, ST-segment elevation myocardial infarction; TIMI, thrombolysis in myocardial infarction

### Echocardiographic evaluation

**Fig. 2:**
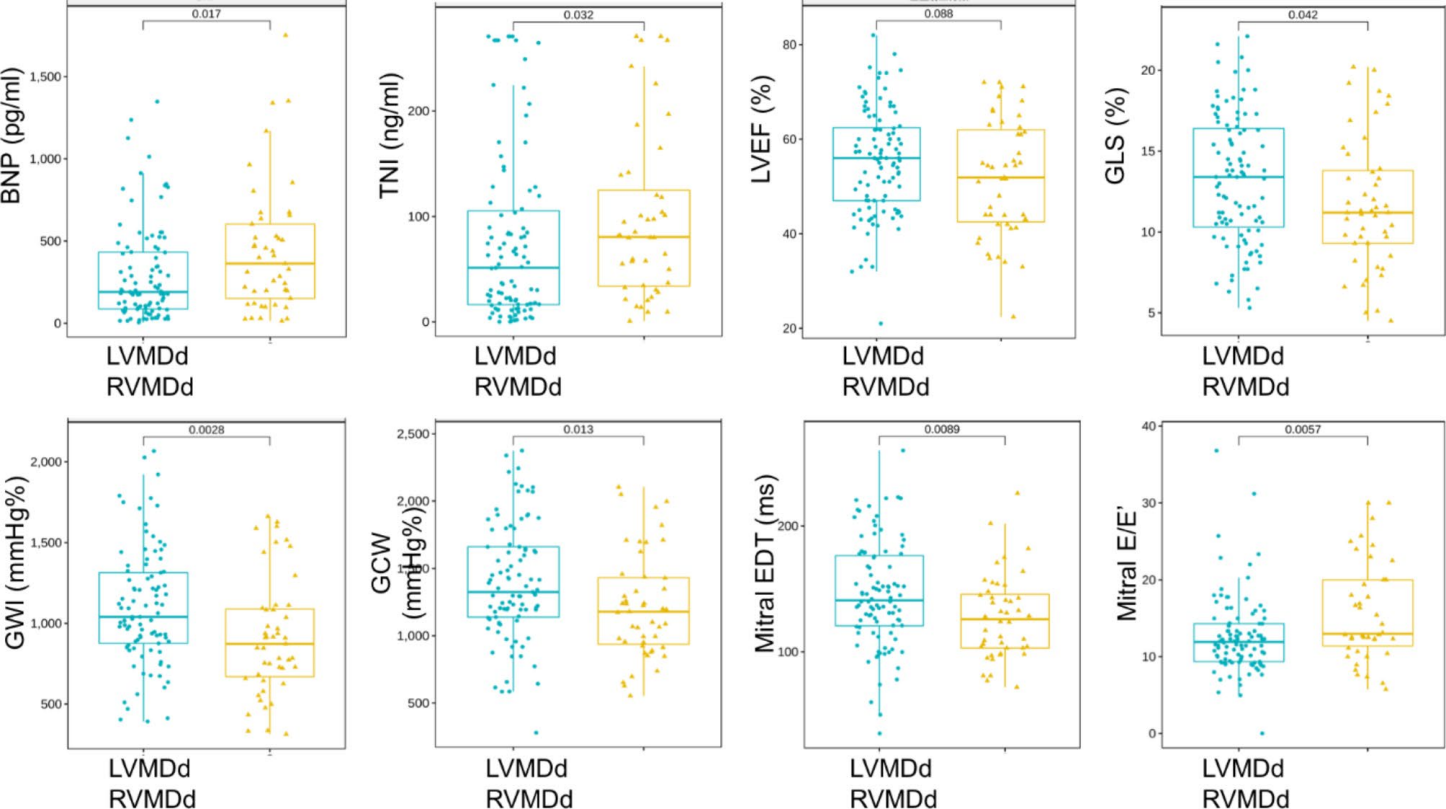
Scatterplots and boxplots diagram of the variables with differences between LVMDd and RVMDd. BNP, brain natriuretic peptide; EDT, deceleration time; GCW, global constructive work; GLS, global longitudinal strain; GWI, global work index; LVEF, left ventricular ejection fraction; LVMDd, left ventricular diastolic mechanical delay; RVMDd, right ventricular diastolic mechanical delay; TNI, troponin I

The echocardiographic characteristics and global values of the LVMW indices between the two groups were shown in Table 3. There were no significant differences in LVEF, RV functional indices and left ventricular or atrial volume between the two groups. But the patients in the RVMDd group exhibited a significantly lower GLS (absolute value 11.7 ± 4.1% vs. 13.2 ± 4.0%, *P* = 0.035), GWI (913.8 ± 365.9 vs. 1098.9 ± 358.8 mmHg%, *P* = 0.005) and GCW (1218.6 ± 392.8 vs. 1393.7 ± 432.7 mmHg%, *P* = 0.021) when compared with those in the LVMDd group. Moreover, two important indicators of diastolic function also showed significant differences: EDT significantly decreased (127.4 ± 33.5 vs. 145.6 ± 41.7 ms, *P* = 0.012), and E/E’ significantly increased [13.0(11.0–20.0) vs. 11.9(9.3–14.3), *P* = 0.006] in the RVMDd group. Figure 2 showed the scatterplots and boxplots diagram of indicators with differences between LVMDd and RVMDd.

**Table 3:** Echocardiographic characteristics

	RVMDd (n = 47)	LVMDd (n = 100)	*P*
BSA (m^2^)	1.9 ± 0.2	1.8 ± 0.2	0.006
SBP at echo (mmHg)	110.3 ± 16.7	116.2 ± 17.4	0.115
DBP at echo (mmHg)	67.9 ± 11.5	70.9 ± 13.2	0.281
LVEDVi (ml/m^2^)	56.6 ± 13.9	55.4 ± 13.6	0.616
LVMI (g/m^2^)	99.3 ± 24.2	98.3 ± 24.3	0.822
LVEF (%)	51.7 ± 12.4	55.4 ± 11.3	0.079
LV GLS (%)	-11.7 ± 4.1	-13.2 ± 4.0	0.035
LAVi (ml/m^2^)	30.2 ± 10.5	29.0 ± 8.4	0.490
Mitral EDT (ms)	127.4 ± 33.5	145.6 ± 41.7	0.012
Mitral E/E’	13.0(11.0–20.0)	11.9(9.3–14.3)	0.006
GWI (mmHg%)	913.8 ± 365.9	1098.9 ± 358.8	0.005
GCW (mmHg%)	1218.6 ± 392.8	1393.7 ± 432.7	0.021
GWW (mmHg%)	191.4 ± 111.0	187.6 ± 99.5	0.836
GWE (%)	83.2 ± 7.7	85.6 ± 7.1	0.071
RAVi	20.3 ± 7.9	19.5 ± 8.0	0.565
TAPSE	2.0 ± 0.4	2.0 ± 0.4	0.266
RVS’	10.2 ± 2.3	10.0 ± 2.3	0.707
RVMPI	61.3 ± 31.4	60.3 ± 30.9	0.874
RVFW GLS	-20.6 ± 5.7	-21.7 ± 6.0	0.306

BSA, Body surface area; DBP, diastolic blood pressure; EDT, deceleration time; GCW, global constructive work; GLS, global longitudinal strain; GWE, global work efficiency; GWI, global work index; GWW, global wasted work; LAVi, left atrial volume index; LV, left ventricle; LVEDVi, left ventricular end-diastolic volume index; LVEF, left ventricular ejection fraction; LVMDd, left ventricular diastolic mechanical delay; LVMI, left ventricular mass index; RAVi, right atrial volume index; RVFW, right ventricular free wall; RVMDd, right ventricular diastolic mechanical delay; RVMPI, RV myocardial performance index; RVS’, RV peak systolic myocardial velocity; SBP, systolic blood pressure; TAPSE, tricuspid annular plane systolic excursion;

### Variables associated with RVMDd

Logistic regression analysis was performed to identify predictors of RVMDd. Variables with P < 0.1 in univariate analysis were subsequently entered into the multivariate model. Clinical data included age, BMI, history of hypertension, SBP at admission, Killip classification, TnI, BNP, and left anterior descending (LAD) coronary artery as the culprit vessel, and echocardiographic data included LVEF, GLS, mitral E/E’, GWI, GCW and GWE. A comprehensive multivariable analysis including clinical and echocardiographic data was performed and the results were presented in Table 4. Only age (odds ratio [OR]: 0.920; *P* = 0.001), BNP (OR: 1.002; *P* = 0.036) level and mitral E/E’ (OR: 1.187; *P* = 0.003) were independently associated with RVMDd.

**Table 4:** Multivariate logistic regression analysis of variables associated with RVMDd

Variables	Wald	*P* value	OR	95%CI
Age	11.711	0.001	0.920	0.977 − 0.965
BNP	4.393	0.036	1.002	1.000-1.003
Mitral E/E’	9.067	0.003	1.187	1.062–1.327

## Discussion

In this study, we explored the ventricular diastolic mechanical sequence in patients with STEMI. LVMDd, an indicator of earlier RV filling than LV filling, was found in 65.8% of patients, whereas RVMDd, an indicator of later RV filling than LV filling, was found in 30.9% of patients. Compared with patients with LVMDd, those with RVMDd had a more severe condition: a lower percentage of Killip I classification and higher values of TnI and BNP levels. Moreover, the incidence of complications in the hospital was substantially higher in patients with RVMDd than in those with LVMDd.

Our previous study confirmed a highly consistent diastolic filling pattern (LVMDd) in the normal population, while the fraction of RVMDd increased significantly in patients with HF [10]. The ventricular wall is much thicker in the LV than in the RV, which may induce LV repolarization to last longer. Previous studies have shown a positive correlation between repolarization time and wall thickness [14, 15]. Myocardial injury in patients with STEMI can likely affect repolarization. In our study, compared with patients with LVMDd, those with RVMDd presented with more severe myocardial dysfunction, as assessed by echocardiography. Patients with RVMDd exhibited a significant decrease in GLS, GWI, and GCW, although there was no significant difference in LVEF. In addition, there was no difference in RV systolic function, suggesting that the occurrence of RVMDd was not related to RV dysfunction. GLS and LVMW indices have been proven to be early indicators of myocardial dysfunction in various diseases [16–20]. Consistent with this finding, patients in the RVMDd group experienced more in-hospital complications. Thus, the occurrence of RVMDd in patients with STEMI may indicate that more attention should be paid to these patients to prevent adverse events.

Our data suggested that age and BNP level were independently associated with RVMDd. In STEMI patients, increased age and BNP level were related to a more severe condition, consistent with the idea that the occurrence of RVMDd might be an indicator of more severe myocardial dysfunction. In addition, E/E’ was significantly increased in patients with RVMDd and independently associated with RVMDd presence. Echocardiographic E/E’ was an important indicator of ventricular diastolic function [21]. Left ventricular diastolic dysfunction usually emerges before systolic dysfunction. Our study suggested that RVMDd might be associated with more severe LV dysfunction in STEMI patients before a significant decrease in LVEF. Further studies should be performed to evaluate the development of inter-ventricular mechanical dyssynchrony after treatment and its influence on the long-term prognosis of patients with STEMI.

The coordinated contraction and relaxation of the left and right ventricles are essential for global heart performance. Asynchronous ventricular mechanical activation in various disease states affects the overall ventricular pump function due to inter- and intra-ventricular asynchrony. At present, the medical treatment of resynchronization mainly focuses on systolic synchrony, such as CRT treatment of heart failure after myocardial infarction. The synchronization in diastole is rarely concerned. But the coronary blood flow perfusion is mainly achieved in diastole. Therefore, the presence of RVMDd may affect the LV myocardial perfusion in STEMI patients. Inter-ventricular dyssynchrony assessment is critical to optimize inter-ventricular delays (V-V delays) in CRT settings. Optimal timing of V-V delays improves ventricular filling capacity and stroke volume, resulting in the reversal of LV remodeling and an improved prognosis [22, 23]. Acute myocardial infarction alters RV and LV mechanical sequences, especially in patients with HF. An abnormal ventricular mechanical sequence monitored by echocardiography should be performed in patients with STEMI as a guide to optimize therapy regimens.

Our study was limited by the relatively small number of participants included and the lack of follow-up data. We could not determine the long-term prognostic value of this inter-ventricular mechanical sequence in STEMI patients. QRS durations according to both groups were not collected for either group. The related variables and prognostic values of the severity of inter-ventricular mechanical delay required further investigation.

## Conclusions

Our study explored the mechanical diastolic sequence of the left and right ventricles in STEMI patients. RVMDd occurrence was associated with more severe impairments in myocardial function and increased total complications in the hospital. Mechanical dyssynchrony assessed by echocardiography is a simple but potential tool to detect early myocardial performance impairment and should be considered for monitoring therapeutic response during routine follow-up in these patients.

## Data Availability

The datasets used and/or analysed during the current study are available from the corresponding author on reasonable request.

## Abbreviations

AUC: areas under the curve
BMI: body mass index
BNP: brain natriuretic peptide
BSA: Body surface area
CAD: coronary artery disease
CKD: chronic kidney disease
CRP: C-reactive protein
DBP: diastolic blood pressure
EDT: mitral deceleration time
GCW: global constructive work
GLS: global longitudinal strain
GWE: global work efficiency
GWI: global work index
GWW: global wasted work
LAD: left anterior descending artery
LAVi: left atrial volume index
LCx: left circumflex artery
LV: left ventricle
LVEDVi: left ventricular end-diastolic volume index
LVEF: left ventricular ejection fraction
LVMDd: left ventricular diastolic mechanical delay
LVMI: left ventricular mass index
MCE: myocardial contrast echocardiography
MW: myocardial work
PCI: percutaneous coronary intervention
RCA: right coronary artery
ROC: receiver operating curve
RVMDd: right ventricular diastolic mechanical delay
SBP: systolic blood pressure
STE: speckle tracking echocardiography
STEMI: ST-segment elevation myocardial infarction
TIMI: thrombolysis in myocardial infarction
TNI: troponin I
